# Knowledge translation opportunities in allergic disease and asthma

**DOI:** 10.1186/1710-1492-6-S4-A2

**Published:** 2010-12-10

**Authors:** Diana Royce

**Affiliations:** 1 AllerGen NCE Inc, Mc Master University, Hamilton, Ontario, L8S 4L8, Canada

## 

AllerGen NCE Inc., the Allergy, Genes and Environment Network, is a national, multi-disciplinary, multi-sectoral network for research and discovery, knowledge translation and capacity building. AllerGen is dedicated to improving the quality of life for allergy asthma and related immune disease sufferers by supporting research that leads to new diagnostic tests, better medications, more effective public policies and an increase in the number of medical professionals researching and practicing in this area. The Networks of Centres of Excellence (NCE) program, of which AllerGen is a part, is a strategic initiative aligned with Canada’s Science and Technology strategy. The NCE program aims to close the ‘development-to-delivery’ gap, and accelerate the rate at which research results contribute to new, evidence-based, cost-effective policies, products and services that generate social and economic benefits for Canadians.

The burden of allergy, asthma and related immune disease is significant and growing world-wide, and while the underlying causes of these diseases are actively being studied, the origins of these diseases are still not well understood. According to the results of the International Study of Asthma and Allergies in Childhood (ISAAC) Study, Phase III (2003) results, 47% of Canadian children have suffered from allergic rhinitis; 39% have experienced wheezing; 22.4% have been diagnosed with asthma; and, 19% have experienced atopic eczema [1]. According to Health Canada, non-food allergies are now the most common chronic condition in Canadians 12 years of age and older [2].

The economic impact of these diseases in Canada is in excess of $15 billion annually, when one includes the cost of ambulatory care, in-patient stays, emergency department visits, physician and facility payments, prescribed medications and productivity losses at school, work and at home as a direct result of these diseases [3]. This annual cost is comparable to the economic impact of arthritis and other chronic conditions. Ontario data show that 14% of all asthma-related emergency department visits occur in children between birth and 4 years of age, and that 21% of asthma prevalent cases were children and adolescents up to 19 years of age [4]. However, hospital admissions for asthma have decreased for both children and adults since 1996, and asthma as a cause of death is relatively uncommon and decreasing among all age groups in the developed world.

Globally, asthma is more prevalent among the developed countries and in major city centres [5]. Among the countries with somewhat lower prevalence rates, such as India and China, which represent 37% of the global population, recent research suggests that as these countries industrialize, allergy, asthma and related immune disease prevalence rates are rising rapidly, mirroring the experience of more developed countries.

Given the Canadian Institutes of Health Research’s strategic vision to position Canada as a world leader in the creation and use of knowledge derived from health research that benefits Canadians and the global community, Canadian researchers and their international partners have a significant opportunity to work in collaborative networks to accelerate the translation of *research into practice*, and *knowledge to action*, to improve allergic disease and asthma awareness, education, management and control.

A recent analysis by Teresa To, from The Hospital for Sick Children [6], reveals that for Ontarians, the lifetime risk of developing chronic asthma is 1 in 3 - the same as the risk of developing cancer and diabetes. However, unlike cancer and diabetes, the substantial lifetime risk of asthma begins at an early stage in life and persists throughout the life span, triggering heightened disease burden, potential productivity loss and other economic costs.

Building upon the work done in 2004 by a team led by Rejean Landry, AllerGen developed a publicly available KT planning tool called *Knowledge Translation Planning Tools for Allergic Disease Researchers.* This tool provides a guide for researchers, their stakeholders and partners to collaboratively develop translational strategies and tactics that will help accelerate the rate of dissemination, uptake and application of allergy, asthma and related immune disease research to improve the quality of life for patients, facilitate optimal care by health providers and reduce the economic drag resulting from the burden of these diseases [7]. Working with national and international partners, such as the Karolinska Institute in Sweden, AllerGen is committed to facilitating efforts to improve allergic disease and asthma management and control, discover the root causes of these diseases and accelerate the application of research findings and KT activities for social and economic benefits nationally and globally.
